# Moderate-to-Vigorous Physical Activity and Response Inhibition Predict Balance in Adults with Attention Deficit/Hyperactivity Disorder

**DOI:** 10.3390/jcm13040968

**Published:** 2024-02-08

**Authors:** Mansour M. Alotaibi, Robert W. Motl, Despina Stavrinos, Scott W. Snyder, Harshvardhan Singh, Donald H. Lein

**Affiliations:** 1 Department of Rehabilitation, College of Medical Sciences, Northern Border University, Arar 73213, Saudi Arabia; 2 Center for Health Research, Northern Border University, Arar 73213, Saudi Arabia; 3 Department of Kinesiology and Nutrition, University of Illinois, Chicago, IL 61820, USA; robmotl@uic.edu; 4 Department of Psychology, University of Alabama, Tuscaloosa, AL 35487, USA; dstavrinos@ua.edu; 5 Department of Human Studies, University of Alabama at Birmingham, Birmingham, AL 35233, USA; ssnyder@uab.edu; 6 Department of Physical Therapy, University of Alabama at Birmingham, Birmingham, AL 35233, USA; hsingh@uab.edu (H.S.); dlein@uab.edu (D.H.L.J.)

**Keywords:** ADHD, physical activity, postural sway, executive function, response inhibition

## Abstract

**Background:** Some evidence indicates that adults with attention deficit hyperactivity disorder (ADHD) may have balance impairments. This study examined the associations between moderate-to-vigorous physical activity (MVPA), response inhibition (RI), and static balance in this population while off and on psychostimulant medication (PS). **Methods:** Participants (*n* = 40; 30 females; *M* age = 29.0; SD = 6.3 years) wore an ActiGraph GT9X–link around their waist to estimate MVPA levels (minutes/day). To assess RI, participants completed the Delis–Kaplan Executive Function System (D–KEFS) subtests Trail-Making Test (TMT) and Color–Word Interference Test (CWIT). To evaluate static balance, participants completed postural sway area (cm^2^) assessments in four conditions: feet-apart eyes-open (FAEO), feet-apart eyes-closed (FAEC), feet-together eyes-open (FTEO), and feet-together eyes-closed (FTEC). Participants also completed the single-leg standing tests (seconds) with eyes open (SLEO) and with eyes closed (SLEC). **Results:** When off medication, MVPA significantly predicted SLEC (*β* = 0.30; *p* = 0.017). MVPA and TMT significantly predicted FTEO, explaining ~19% of the variance in FTEO; both MVPA and TMT were significant predictors (*β* = –0.33, *p* = 0.027 and *β* = –0.31, *p* = 0.039, respectively). When on medication, TMT significantly predicted FAEC (*β* = 0.17; *p* = 0.047). **Conclusions:** MVPA and RI may be effective parameters in predicting static balance in adults with ADHD when off medication only.

## 1. Introduction

Attention deficit hyperactivity disorder (ADHD) is a neurodevelopmental disorder with three subtypes: predominately inattentive, predominately hyperactive–impulsive, and combined [1]. ADHD is commonly thought to be a childhood condition [2], but it may persist into adulthood [3]. Approximately 1.0% [4] to 4.4% [3] of American adults aged 18–54 years have a diagnosis of ADHD, with males diagnosed two times more frequently than females [3]. Adults with ADHD have high rates of falls in one year compared to adults without ADHD (Adult ADHD, 27 falls; controls, 0 falls) [5]. This increased rate of falls could be associated with balance impairments, as observed in children [6] and adults [7] with ADHD. 

Psychostimulant medications (PS), including methylphenidate (MPH) and amphetamine (AMP), are commonly prescribed to treat ADHD symptoms [8]. PS use may improve balance outcomes in children [6,9,10] and has been associated with improved balance outcomes in adults with ADHD [11]. The mechanism of how PS may improve balance outcomes is not well understood. One potential mechanism is that these medications upregulate dopamine concentrations in the prefrontal cortex and basal ganglia [12,13]. These areas of the brain are involved in controlling motor tasks, including maintaining upright posture [14]. Notably, physicians recommend the practice of medication holidays for adults with ADHD [15], and adults with ADHD often ask for medication holidays or to be off medications, specifically during weekends and vacations [15], thereby posing a greater risk of balance-related injuries during unmedicated periods [7]. 

Moderate-to-vigorous physical activity (MVPA) may be associated with improved balance in adults with ADHD. Several forms of MVPA, like resistance and aerobic exercises, are used for improving balance and preventing falls in older adults, likely by stimulating several components of physical health, such as cardiovascular fitness, balance, and muscle strength [16,17]. These forms of MVPA may also improve balance in adults with ADHD through the same mechanisms, but no studies have examined the effect of such MVPA forms on balance in adults with ADHD. Additionally, MVPA significantly predicted postural sway performance in young adults without ADHD [18], but we found no articles concerning the relationship between MVPA and balance in adults with ADHD. Information on the relationship between MVPA and balance could be imperative to understanding the risk of injury during physical activities. Specifically, we do not know if these relationships are affected by the medication status of adults with ADHD. Thus, engaging in regular MVPA may be associated with improved balance in this population whether off or on PS, and could be a potential intervention for improving static balance.

Improving response inhibition (RI) may also help enhance static balance in adults with ADHD. RI is the capacity to withhold a response or override an ongoing response which affects ADHD symptoms like planning and problem-solving [19]. Decreased RI may thus disrupt motor planning, and therefore may be a factor that is correlated with balance performance in adults with ADHD. Moreover, the cerebellum is involved in executive functioning, including RI [20]. This part of the brain also regulates sensorimotor function, needed for balance, via direct cerebellar projections towards the parietal cortex [21]. Cerebellar volume and activity are reduced in adults with ADHD compared to controls [22], which may contribute to impairments in RI and balance performance. Specifically, cerebellar volume is associated with increased postural sway, indicative of poor static balance in adults with ADHD when off their medication [7]. Thus, RI may be associated with balance performance in adults with ADHD. Nevertheless, little information is known regarding this relationship in this population when off or on their PS. Such information could provide insight into the role of RI as a possible factor affecting balance performance in adults with ADHD regardless of PS status.

To date, researchers have not examined the associations between MVPA levels, RI, and static balance performance in adults with ADHD when off or on PS medications, yet determining those associations could help improve our understanding of factors influencing balance performance in this population. Specifically, determining predictors of balance performance in adults with ADHD could be investigated further as possible interventions to improve balance and decrease balance-related falls. Therefore, this study investigated the association of MVPA and RI with static balance performance in adults with ADHD when off and on PS medications. We hypothesized that MVPA levels and RI performance would be significantly correlated with and predict static balance performance when off or on PS.

## 2. Materials and Methods

### 2.1. Participants

Prior to participating in the study, participants provided written informed consent, approved by the Institutional Review Board (Protocol number: IRB–300006200) at the University of Alabama at Birmingham (UAB) on 21 March 2021. Participants were recruited by posting fliers around UAB, advertising in the UAB eReporter (https://www.uab.edu/reporter/ accessed on 15 July 2021), and sending email invites to potential participants using the UAB Informatics for Integrating Biology and the Bedside (i2b2) data. To be enrolled in this study, participants had to be (a) aged 20–55 years, (b) diagnosed with ADHD by a physician or a psychologist, (c) prescribed with MPH- or AMP-based PS to control ADHD symptoms for a minimum of three months [10], (d) free of major orthopedic, cardiovascular, neurological, or respiratory diseases, (e) able to speak and read English proficiently, and (f) have participated in free-living physical activity in the community. Exclusion criteria included the use of assistive devices for mobility, difficulties following instructions, and the use of medications that affect movement regulation and neural firing excitability, such as antipsychotics or anticonvulsants.

Forty-five participants enrolled in this study, but five participants were excluded from the analysis based on incomplete data. The final sample included 40 adults with ADHD [30 females (75.0%); *M* age = 29.0 *SD* = 6.3 years]. Most participants were diagnosed with the inattentive subtype (*n* = 13; 32.5%) or combined subtype (*n* = 11; 27.5%) of ADHD, used AMP-based stimulants (*n* = 35; 87.5%), were Caucasian (*n* = 30; 75.0%), were involved in graduate studies or had received a graduate degree (*n* = 22; 55.0%). On average, participants were slightly overweight (*M* BMI = 28.0 ± 7.7) (Table 1). Only seven participants met the minimum required time of 150 min/week of MVPA as suggested by PAG [23].

### 2.2. Procedures

**Figure 1:**
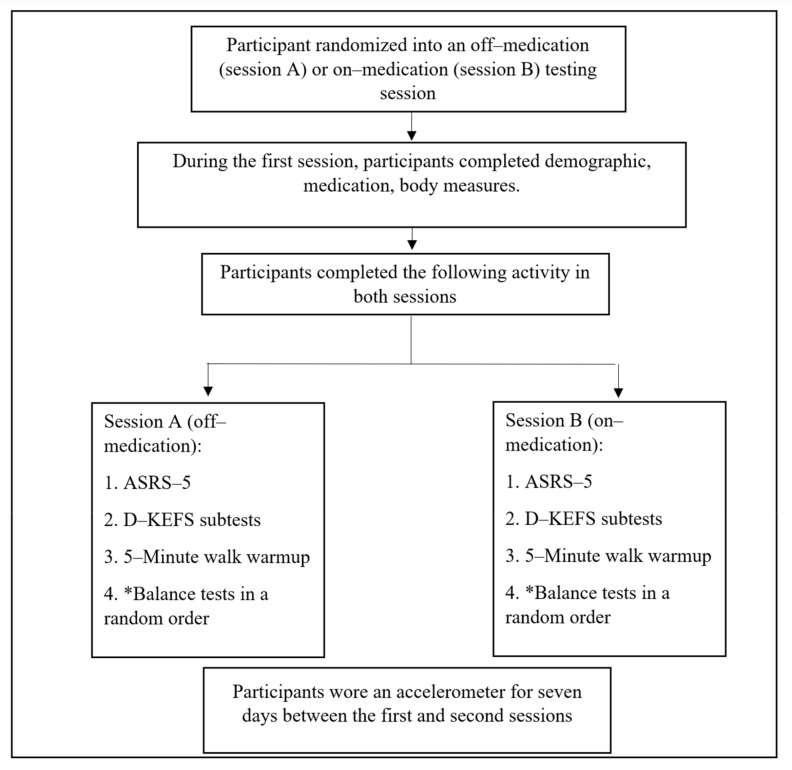
Summarized study procedures. Note: ASRS–5: Adult Self-Report Rating Scale–5; D–KEFS: Delis–Kaplan Executive Function System. * (1) Standing with feet apart with eyes open, (2) standing with feet apart with eyes closed, (3) standing with feet together with eyes open, (4) standing with feet together with eyes closed, (5) single leg standing test with eyes open, (6) single leg standing test with eyes closed.

This study was part of a larger study that examined the effects of psychostimulant medications on static balance performance in adults with ADHD [11]. Data collection for this cross-sectional study occurred in the UAB Human Performance Laboratory between May 2021 and February 2022. Participants visited the laboratory twice with a range of one to four weeks scheduled between the two sessions, and this varied based on the academic schedules and demands of the participants. Participants were randomly assigned (https://www.random.org/ accessed on 15 July 2021) to come to the first session off or on PS. For the off-medication session, investigators directed the participants to skip PS medication 24 h before data collection to ensure no systematic PS effects on the central nervous system [24,25]. For the on-medication session, investigators instructed the participants to use PS medication as prescribed by their treating physician. All participants reported being on or off their assigned medication by answering a self-reported questionnaire during each session. In both sessions, participants completed (a) a questionnaire (demographics, psychostimulant medication use information, other medication use, and ADHD symptoms), (b) two Delis–Kaplan Executive Function System (D–KEFS) subtests (i.e., trail-making test [TMT] and color–word interference test [CWIT]), (c) body mass and height measurements, and (d) balance tests. Body height and body mass measurements were collected during the first visit. In addition, participants were given an accelerometer, along with instructions, to measure physical activity for seven days at the completion of the first session to wear between the first and second sessions while taking their medication as prescribed by their physicians. The non-dominant leg was used for balance tests and the side to place the accelerometer. The non-dominant leg was decided by asking participants, “Which leg do you kick a ball with?” (Figure 1).

### 2.3. Outcome Measures

#### 2.3.1. Demographic and Anthropometrics

Age, sex, race, ADHD medication information, and educational level were collected via a customized questionnaire. ADHD subtype was confirmed by a physician’s or psychologist’s report. Investigators collected body mass (kg) and body height (cm) by using, respectively, a scale (Garmin Ltd., Southampton, UK) and a stadiometer (Charder HM200P Stadiometer, Taichung City, Taiwan). BMI was calculated by dividing mass by body height squared (kg/m^2^).

#### 2.3.2. ADHD Symptoms

Investigators used the updated version of the World Health Organization Adult ADHD Self-Report Screening Scale for DSM–5 (ASRS–5) to measure participants’ ADHD symptoms and to determine PS effects on ADHD symptoms. The ASRS–5 is a 6-item questionnaire adopted from the Composite International Diagnostic Interview for DSM–5 (CIDI–5.0) for adults aged 18 years or older [26]. The ASRS–5 has excellent sensitivity (91.4%), specificity (96.0%), and area under the curve (AUC = 0.94) in detecting ADHD symptoms [26]. Participants completed this questionnaire when off and on their medication. Investigators scored this questionnaire using the proprietary scoring rules for the DSM–5 version with permission from New York University and Harvard University. 

#### 2.3.3. Physical Activity Accelerometry Measure

Between the two testing sessions, participants received instructions and demonstrations on how to wear a small (3.5 × 3.5 × 1 cm) and lightweight (14 g) accelerometer device (ActiGraph GT9X link, ActiGraph, LLC., Pensacola, FL, USA) secured with a waistband belt clip near the anterior superior iliac spine (ASIS) of the non-dominant side [27]. Participants wore the device for seven days, starting the day after the first testing session, and during the waking hours of the day. The GT9X link accelerometer measures movement by generating an electrical signal proportional to the force acting on it along three orthogonal axes (tri-axial) and provides more accurate estimates of movement if worn near the ASIS [28]. Raw data were post-processed using ActiLife 6 software (ActiLife, ActiGraph, LLC., Pensacola, FL, USA). Data were expressed as counts per epoch (epoch length was 60 s, sample frequency was 30 Hz) to quantify physical activity data of at least one valid day (i.e., ≥10 h of wear time) for more accurate physical activity estimates [29,30]. The Troiano’s cut-off points in counts per minute (CPM) classified physical activity levels as follows: 0–99 CPM range indicates sedentary behavior, 100–2019 CPM range indicates light physical activity (LPA), and ≥2020 CPM range indicates MVPA [30]. The derived physical activity outcome used in this study was average minutes/day of MVPA [18]. MVPA level showed excellent test–retest reliability (intraclass correlation; ICC = 0.83) in adults without ADHD using a GT9X device [31].

#### 2.3.4. RI–Delis–Kaplan Executive Function System

The D–KEFS employs nine subtests that comprehensively measure high-level cognitive function and frontal lobe integrity [32]. Two subtests were employed for the purpose of this study to assess RI [32]: (1) the Trail Making Test (TMT) and (2) the Color–Word Interference Test (CWIT). While the TMT consists of 5 conditions, we only used the results from the TMT Number–Letter Switching (condition 4) since it primarily measures RI [32]. Two out of the four conditions of the CWIT were used in the study analyses to measure RI: Inhibition and Inhibition/Switching subtests (conditions 3 and 4) [32]. Prior to testing participants’ RI, participants completed a series of tests (e.g., number sequencing and naming color) to ensure that they met sufficient lower cognitive skill levels to complete the RI testing. Participants completed these two D–KEFS subtests in both sessions (off- and on-medication) because improvements in executive function delivered by psychostimulant medications may account for improving motor performance [33,34]. The D–KEFS scaled scores for each subtest were determined by following the technical manual guidelines [32] and using PsychCropCenter software 2.0.1 (Harcourt Assessment, Inc., San Antonio, TX, USA). These tests showed good psychometric properties in adults with ADHD [32]. Higher D–KEFS scores represent better RI performance.

#### 2.3.5. Postural Sway Measurements and Single-Leg Standing Tests

Participants performed a 5 min walk on a treadmill at their self-selected speed and without an incline as a controlled warm-up, before undergoing balance testing. The Borg Scale for Perceived Exertion [35] cued participants to maintain light exertion when walking on the treadmill. Investigators instructed and monitored the participants to keep a score between 6 and 11, which indicates light exertion [35]. Participants performed all static balance tests on a force platform (1000 Hz, AMTI, Watertown, MA, USA). Participants underwent four different tests to measure sway area. Each test lasted 30 s and included standing with feet shoulder width apart with (1) eyes open (FAEO) and (2) eyes closed (FAEC); and standing with feet together with (3) eyes open (FTEO) and (4) eyes closed (FTEC). Prior to data collection, the physical therapist demonstrated each test condition and after each demonstration asked the participants to demonstrate the balance test to assess understanding and safety. These test conditions demonstrated sensitivity to balance performance when comparing adults with ADHD with a control [7]. These tests are also reliable and valid in measuring static balance in adults without ADHD [36]. Software written in MATLAB R2020a (MathWorks Inc., Natick, MA, USA) was used to process postural sway data. The Center of Pressure (COP) trajectory was analyzed for 20 s, eliminating the first and last 5 s of each trial for additional accuracy [11,37]. A 12Hz low-pass Butterworth filter (4th order) was applied during data extraction. The calculated variable was sway area (cm^2^) for each test condition. Postural sway calculations were performed using postural sway equations provided by Doyle et al. [38], using the 95% confidence ellipse area based on anteroposterior and mediolateral standard deviation (SD) of displacement.

Participants also completed two other balance tasks: (1) a single leg test on a firm surface with eyes open for 30 s (SLEO), and (2) a single leg test on a firm surface with eyes closed for 30 s (SLEC). Participants placed their hands on their waists during each test. An experienced and licensed physical therapist provided instructions and guarded participants during the tests. If a participant touched the floor with the non-weight-bearing foot, the examiner recorded the time (i.e., touch time [s]) and asked the participant to resume the testing position. The timer was kept going as they repositioned themselves to the test position. If they lost balance when performing SLEC, they were instructed to open their eyes, resume the testing position, and then close their eyes again. Participants were familiarized with all balance conditions through a demonstration by the physical therapist and then demonstrated the test before data collection. The single-leg tests showed excellent test–retest reliability in adults without ADHD [39]. The postural sway and balance test order was randomized to minimize the ordering effect.

### 2.4. Statistical Analyses

Statistical Package for the Social Sciences software v27.0 (SPSS; IBM Corp., Armonk, NY, USA) was used for all statistical analyses. Means and standard deviation (SD) were used to summarize age, body height, body mass, BMI, ASRS–5, MVPA level, TMT–Number Letter Switching score, CWIT Inhibition score, CWIT Inhibition/Switching score, and balance outcome measures (SLEC time, as well as FAEO, FAEC, FTEO, and FTEC sway areas). Frequencies were used to summarize sex, race, education level, dominant leg, ADHD subtype, and ADHD medication type. 

The following variables of interest were chosen for exploratory correlation analysis and based on our a priori defined hypothesis that balance variables [sway area of FAEO, FAEC, FTEO, and FTEC sway areas; and SLEC time (s)] will be correlated with physical activity (MVPA; minutes/day) and RI (TMT: Number–Letter Switching and CWIT (Inhibition and Inhibition/Switching)). In addition, correlation analyses were performed between postural sway area, body mass, and body height to examine whether anthropometric variables may have been associated with postural sway measurements. For the multiple correlation analysis, we used an alpha level of 0.05 to determine significant correlations [40,41]. Next, significantly correlated variables of interest were included in multiple linear regression models. 

Skewness statistics and box plots indicated no violation of the normality assumption of each variable entered in the analyses. Pearson correlations were used to examine the correlations between the variables of interest: MVPA level, TMT score, CWIT score, postural sway areas, and SLEC time. Correlation coefficients were interpreted as weak correlation (0.1–0.3), moderate correlation (0.3–0.5), and strong correlation (>0.5) [42]. These correlations yielded three regression models: Model 1 (off medication): the predictor was MVPA level, and the dependent variable was SLEC. Model 2 (off medication): the predictors were MVPA level and TMT Number–Letter Switching and the dependent variable was FTEO sway area. Model 3 (on medication): the predictor was TMT Number–Letter Switching, and the dependent variable was the postural sway area during FAEC. Multi-collinearity was tested for each multivariate linear regression model using the variance inflation factor (VIF) index. Values greater than 5 indicated multicollinearity [43]. An alpha level of 0.05 was the criterion for statistical significance for all statistical tests and model building.

## 3. Results

### 3.1. Associations among the Outcomes: Off-Medication Status

**Figure 2:**
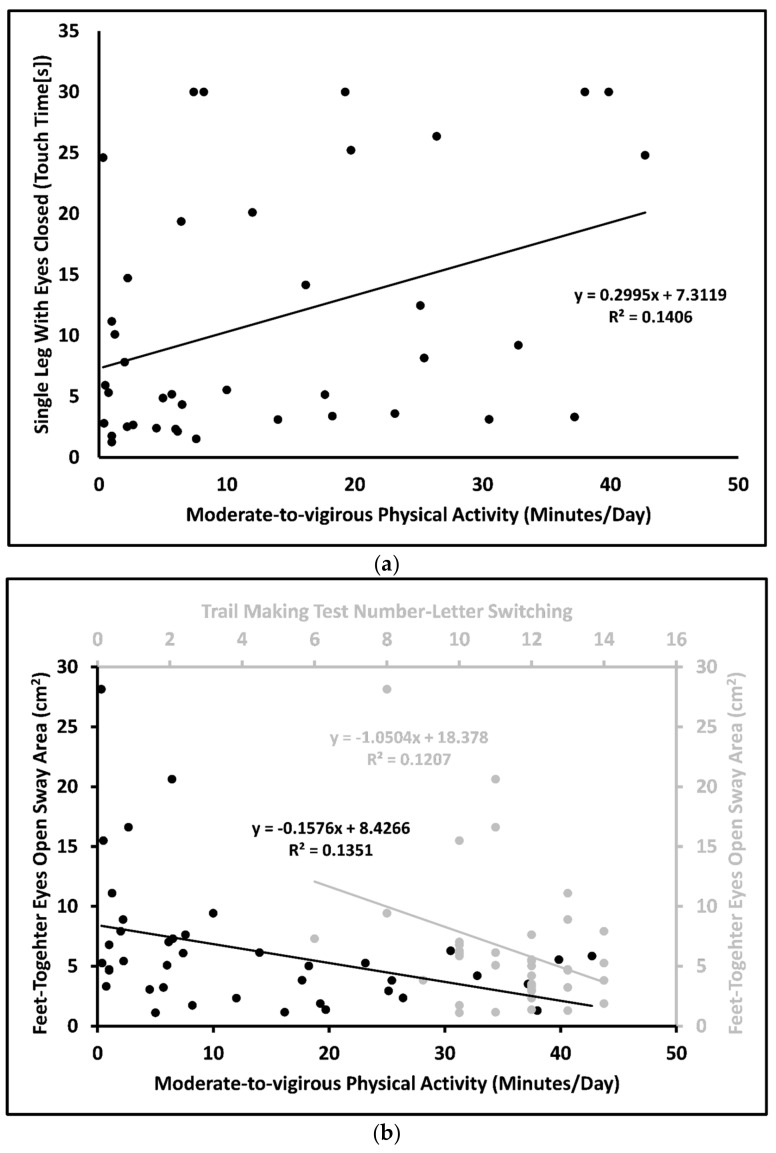
(**a**) Moderate-to-vigorous physical activity plotted against single leg standing balance test on firm surface with eyes closed touch time. (**b**) Moderate-to-vigorous physical activity plotted against and Trial Making Test Number–Letter Switching score plotted against feet together with eyes open balance test during off-medication status.

The correlation analyses identified a significant positive moderate correlation between MVPA and SLEC (*r* = 0.38; *p* < 0.05). Greater engagement in MVPA was correlated with a longer time until touching the floor with the non-weight-bearing leg during SLEC, which suggested better static balance performance (Figure 2a). There was a negative significant moderate correlation between MVPA level and FTEO sway area (*r* = –0.37; *p* < 0.05) and between TMT Number–Letter Switching score (RI) and FTEO sway area (*r* = –0.35; *p* < 0.05), indicating that engagement in MVPA and higher RI scores were correlated with lower sway area (better static balance) (Figure 2b). There were no significant correlations between the other D–KEFS variables or MVPA levels with static balance scores (all *p* > 0.05) (Table 2). There were no significant correlations between body mass, body height, and postural sway variables (*r* < 0.25, *p* > 0.05; Supplementary Table S1). MVPA level significantly predicted SLEC (*β* = 0.30; *p* = 0.017). Further, the multivariate analysis indicated that MVPA level and TMT Number–Letter Switching score significantly predicted FTEO sway area (*F*(1,38) = 5.550; adjusted *R*^2^ = 0.189; *p* = 0.008), explaining about 19% of the variance in FTEO sway area. Both MVPA level and TMT Number–Letter Switching score were significant predictors (*β* = –0.33, *p* = 0.027 and *β* = –0.31, *p* = 0.039, respectively) (Table 3).

### 3.2. Associations among the Outcomes: On-Medication Status

**Figure 3:**
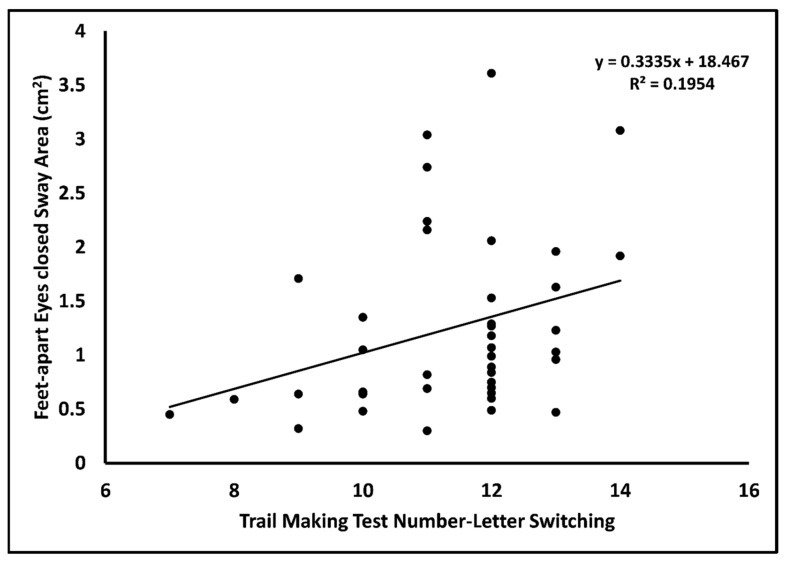
Trail making test number–letter switching plotted against feet apart standing balance test with eyes open.

There was a significant moderate positive correlation between TMT Number–Letter Switching (RI) score and FAEC sway area (*r* = 0.32; *p* < 0.05) (Table 2; Figure 3). No other significant correlations existed between MVPA level and RI variables with static balance scores (all *p* > 0.05). The TMT Number–Letter Switching score significantly predicted the FAEC sway area (*β* = 0.17; *p* = 0.047) (Table 4). There were no significant correlations between body mass, body height, and postural sway variables (*r* < 0.25, *p* > 0.05; Supplementary Table S2).

## 4. Discussion

This study examined associations between MVPA levels and RI with static balance in adults with ADHD. We observed that MVPA and RI moderately and directly correlated with and predicted static balance in adults with ADHD when adults were off PS. These findings indicate that better MVPA levels and RI scores were associated with better static balance performance in this population. In addition, we reported no correlations between MVPA and RI with static balance when participants were on PS, except TMT Number–Letter Switching (an RI measure). This measure of RI was positively associated with and predicted balance when participants were on medication, which indicated that worse RI was associated with poor balance. Thus, our hypotheses were only partially supported in this study. 

Like young adults without ADHD [18], greater MVPA level was associated with improved static balance in adults with ADHD in this study when off their medication. Engaging in MVPA improves the activation of the prefrontal cortex and the somatosensory cortex [44], which might improve balance [45]. Improved static balance during off medication periods may also be due to increased circulating dopamine concentrations which have been found to increase after bouts of resistive exercise in healthy males [46]. An improved dopaminergic effect is thought to improve static balance in children [6]. Like adults without ADHD, balance training is probably not needed in adults with ADHD for them to complete daily tasks and locomotion, but engaging in MVPA was associated with improved balance in adults without ADHD [18]. Engaging in MVPA improved balance in middle-aged adults without ADHD [47]. Our results suggest that adults with ADHD could benefit from programs that include MVPA in addition to their PS medication to improve their static balance when off their PS, especially on their medication holidays (a practice that is common among adults with ADHD) [15]. More studies are warranted to examine if MVPA would result in balance improvements. 

Our results also demonstrated that increased MVPA levels only predicted participants’ static balance performance during off PS, and not when they were on PS. This finding was surprising since a previous study showed that dopamine concentrations increased after a single bout of resistive exercise in healthy males [46]. Increasing levels of dopamine in the prefrontal lobe and basal ganglia, areas of the brain that control the maintenance of upright balance (14), are a potential explanation for how PS helps to improve balance in people with ADHD. We hypothesized that the dopamine produced by greater MVPA could potentially add to the dopamine released by PS and improve static balance in adults with ADHD. In our study, the correlations between MVPA levels with FTEO sway area and SLEC time were approaching significance [r = –0.3, *p* = 0.090; and r = 0.3, *p* = 0.082, respectively] when participants were on PS. Perhaps having only seven adults in this sample who engaged in a minimum of 150 min of MVPA, which is the minimum recommended amount of physical activity as suggested by PAG [23], affected these correlations. This low number of participants meeting PAG recommendations when on PS may have decreased the magnitude of the correlation between MVPA and static balance performance. 

Like MVPA, we found that RI moderately and positively correlated with and predicted static balance in adults with ADHD when on PS. We found no research concerning the relationship between RI and static balance in this population. However, a previous study found positive associations between RI and clinical balance outcomes in people with Parkinson’s disease [48]. Therefore, RI may be important for regulating balance across different populations. Future studies should determine if improving RI could improve balance in different populations at risk of balance impairments, such as adults with ADHD [7]. However, we did not see any correlations between measures of RI and static balance when participants were taking PS. Perhaps since PS improves attention and motor planning in individuals with ADHD, higher levels of RI function do not add to the effects of PS. Regardless, having higher levels of RI may be beneficial to reduce falls when adults with ADHD are on medication holidays, which are frequently used by individuals with ADHD [15].

Surprisingly, improved RI scores were associated with poorer FAEC scores when participants were on PS. However, the effect size was small (*R*^2^ ~8%) and future studies need to be carried out to confirm this relationship. If this relationship persists in other studies, perhaps another explanation exists. The FAEC condition of the postural control test is considered less challenging compared to feet-together conditions and one-leg standing with eyes closed due to the decrease in the base of support and having no vision. A previous study in children with ADHD found that when on PS, children performed significantly better when the balance task became more challenging [6]. Therefore, participants in this study may not have attended to the directions to remain still when performing FAEC since it lacked challenge. 

Future research should examine whether improving MVPA and RI function could improve balance in adults with ADHD. This study suggests that healthcare providers should assess physical activity levels and RI when providing healthcare services to this population, especially when injuries due to falls are being reported by their patients. Implementing strategies to improve MVPA and RI may help with balance impairments in this population, despite the lack of strong evidence. 

This study has a few limitations. First, causal inferences of MVPA and RI on static balance performance should not be made since this is a cross-sectional study. However, since little was found in the literature regarding associations between RI and MVPA with balance performance in this population, the use of this design was appropriate to avoid wasting valuable resources needed to perform random control trials on variables that may not have been correlated or predictive of static balance. While MVPA and RI correlated and predicted balance performance in adults with ADHD when off medication, no variables that we measured predicted static balance when taking PS. Furthermore, MVPA and RI only explained 18% of the variance of balance performance, indicating that other variables may better predict balance in this population when off medication. Nonetheless, MVPA and RI were trending towards being associated with balance when participants were on PS, and the correlations were moderate and predictive when participants were off medication. With more participants, the correlation strength and adjusted R-square values may be higher, thus warranting further study. In addition, caution for the generalization of these findings to other age groups of individuals with ADHD should be taken. For example, the age range in this study included only young and middle-aged adults. Furthermore, the sample in this study was different from typical samples of individuals with ADHD, since more women participated in this study than men [3]. This is probably due to recruiting at a university campus, where women make up a greater proportion of the student population [49]. In addition, the correlation analysis protocol used multiple correlation matrices without correcting for the p-value, which could increase the likelihood of type I error and of finding significant correlations by chance. However, we followed the correlation analysis with multiple linear regression models to confirm the association between the correlated variables found in the correlation analysis with an interpreted effect size, which allowed us to interpret our findings more confidently. Finally, the study design does not allow us to determine if MVPA or RI by itself provides protective effects on balance impairments in adults with ADHD not using PS. Therefore, future research should determine whether MVPA and RI improve balance in the short and long term.

We believe that implementing different forms of MVPA or RI training has very low risk and potentially high benefits, especially since these interventions are being used to improve other ADHD symptoms in adults [50,51]. In addition, we found that our study sample was overweight and the majority of them did not meet PAG guidelines for physical activity. This is consistent with a previous study that showed that adolescents with ADHD did not meet PAG physical activity guidelines [23]. Regular MVPA is often used in combination with diet changes to help adults lose body weight. Thus, finding ways to motivate adults with ADHD to participate regularly in recommended levels of MVPA to help control ADHD symptoms and weight may be helpful regardless of regular MVPA helping to improve balance in adults with ADHD. 

## 5. Conclusions

MVPA and RI domains of executive function significantly predicted static balance in adults with ADHD. However, these relationships were observed with off-medication status only. We believe that regularly engaging in MVPA and RI training (e.g., playing chess) could be recommended to individuals who take PS for ADHD to help improve static balance during medication holidays or when they are off medication. This recommendation is low-risk with potentially high benefit since these activities help with symptom management and regular MVPA is recommended for managing weight. Finally, further research should be performed to determine if MVPA and cognitive behavioral therapy improve balance impairments in adults with ADHD. 

## Figures and Tables

**Table 1:** Demographic and clinical outcomes of participants.

Characteristic	All Participants *n* = 40	
	Mean	SD/%
Age (y)	29.0	6.3
Sex *n* (%)		
Male	10	25.0
Female	30	75.0
Race *n* (%)		
Caucasian	30	75.0
Black or African American	5	12.5
Asian	2	5.0
Mixed Race	3	7.5
Body Mass (kg)	81.8	22.4
Body Height (cm)	170.5	9.4
Body Mass Index (kg/m^2^)	28.0	7.7
Dominant Leg *n* (%)		
Left	5	12.5
Right	35	87.5
Education Level *n* (%)		
Some College Education	5	12.5
Undergraduate	13	32.5
Graduate Level	22	55.0
Psychostimulant Medication *n* (%)		
MPH-based	5	12.5
AMP-based	35	87.5
Adult Self-Report ADHD Scale–5		
Off medication	18.6	2.4
On medication	13.6	4.0

**Table 2:** Pearson correlation coefficients among the variables of interest.

Variable	N	M	SD	1	2	3	4	5	6	7	8	9
Off Medication												
1. SLEC (touch time in seconds)	40	11.3	10.2	—								
2. FAEO (sway area in cm^2^)	40	1.5	2.2	–0.18	—							
3. FAEC (sway area in cm^2^)	40	1.7	1.7	–0.11	0.16	—						
4. FTEO (sway area in cm^2^)	40	6.4	5.5	–0.04	–0.02	0.40 *	—					
5. FTEC (sway area in cm^2^)	40	9.2	5.8	–0.21	0.06	0.46 **	0.52 **	—				
6. MVPA (minutes/day)	40	13.2	12.8	0.38 *	0.01	–0.16	–0.37 *	–0.01	—			
7. TMT Number–Letter Switching	40	11.5	1.8	0.01	–0.21	–0.05	–0.35 *	–0.16	0.11	—		
8. CWIT Inhibition	40	11.3	2.7	0.19	–0.15	0.04	–0.28	–0.11	0.33 *	0.44 **	—	
9. CWIT Inhibition/Switching	40	10.5	2.8	0.03	–0.27	0.03	–0.28	–0.04	0.19	0.34 *	0.72 **	—
On Medication												
1. SLEC (touch time in seconds)	40	13.7	10.8	—								
2. FAEO (sway area in cm^2^)	40	1.4	1.4	0.08	—							
3. FAEC (sway area in cm^2^)	40	1.3	0.8	0.34 *	0.45 **	—						
4. FTEO (sway area in cm^2^)	40	4.6	2.0	–0.17	0.38 *	0.45 **	—					
5. FTEC (sway area in cm^2^)	40	7.6	4.8	0.34 *	0.22	0.43 **	0.25	—				
6. MVPA (minutes/day)	40	13.2	12.8	0.27	–0.18	–0.22	–0.28	–0.08	—			
7. TMT Number–Letter Switching	40	11.4	1.5	–0.17	–0.11	0.32 *	0.14	0.13	0.04	—		
8. CWIT Inhibition	40	11.6	2.0	–0.07	0.16	0.22	0.12	–0.11	0.04	0.25	—	
9. CWIT Inhibition/Switching	40	10.4	2.7	–0.06	0.13	0.23	0.15	–0.14	0.08	0.19	0.72 **	—

Notes: SLEC: single leg on firm surface with eyes closed; FAEO: Feet apart standing balance test with eyes open; FAEC: Feet apart standing balance test with eyes closed; FTEO: Feet together standing balance test with eyes open; FTEC: Feet together standing balance test with eyes closed; MVPA: moderate-to-vigorous physical activity. TMT: Trial Making Test; CWIT: Color–word interference test. * Correlation is significant at the 0.05 level (2-tailed). ** Correlation is significant at the 0.01 level (2-tailed). Bold indicates significant correlation.

**Table 3:** Predictors of balance from multivariate linear regression analyses during off-medication status.

Effect	Estimate	SE	Standardized Beta	95% CI	*p*
A. Outcome SLEC touch time [*F*(1,38) = 6.218, adjusted *R*^2^ = 0.118, *p* = 0.017]					
Intercept	7.312	2.191		2.877, 11.747	0.002
**MVPA**	**0.300**	**0.120**	**0.375**	**0.056, 0.543**	**0.017**
B. Outcome FTEO sway area [*F*(1,38) = 5.550, adjusted *R*^2^ = 0.189, *p* = 0.008]					
Intercept	19.005	5.059		8.754, 29.255	<0.001
**MVPA**	**–0.143**	**0.062**	**–0.334**	**–0.269, –0.017**	**0.027**
**TMT**	**–0.941**	**0.438**	**–0.311**	**–1.829, –0.052**	**0.039**

Note: SLE: Single-leg standing balance test with eyes closed; FTEO: Feet together standing balance test with eyes open: MVPA: Moderate-to-vigorous physical activity; TMT: Trial Making Test: Number–Letter Switching. Bold indicates significant association.

**Table 4:** Predictors of balance from univariate linear regression analyses during on-medication status.

Effect	Estimate	SE	Standardized Beta	95% CI	*p*
Outcome FAEC sway area [*F*(1,38) = 4.213, adjusted *R*^2^ = 0.076, *p* = 0.047]					
Intercept	–0.657	0.938		–2.557, 1.243	<0.001
**TMT**	**0.168**	**0.082**	**0.316**	**0.002, 0.333**	**0.047**

Note: FAEC: Feet apart standing balance test with eyes closed; TMT: Trial making test: number–letter switching. Bold indicates significant association.

## Data Availability

The data presented in this study are available on request from the corresponding author.

## Supplementary Materials

The following supporting information can be downloaded at: https://www.mdpi.com/article/10.3390/jcm13040968/s1, Table S1: Pearson correlation coefficients between balance and anthropometric variables when off-medication. Table S2: Pearson correlation coefficients between balance and anthropometric variables when on-medication.
