# Supplementary material for: Moderate-to-Vigorous Physical Activity and Response Inhibition Predict Balance in Adults with Attention Deficit/Hyperactivity Disorder
Source: J Clin Med. 2024 Feb 8;13(4):968. doi: 10.3390/jcm13040968 (PMC10889301; doi:10.3390/jcm13040968)
Supplement: Supplementary file 1 [file jcm-13-00968-s001.zip › jcm-2841544-supplementary.pdf]

Supplementary Table S1. Pearson Correlation Coefficients Between Balance and Anthropometric Variables When Off-Medication.

Variable	n	M	SD	1	2
1. Body mass (kg)	40	81.8	24.2	—	
2. Body height (cm)	40	170.5	9.4	0.43	—
3. FAEO (sway area in cm^2)	40	1.5	2.2	0.08	0.24
4. FAEC (sway area in cm^2)	40	1.7	1.7	0.06	0.04
5. FTEO (sway area in cm^2)	40	6.4	5.5	0.08	-0.04
6. FTEC (sway area in cm^2)	40	9.2	5.8	0.20	0.04

Supplementary Table S2. Pearson Correlation Coefficients Between Balance and Anthropometric Variables When On-Medication.

Variable	n	M	SD	1	2
1. Body mass (kg)	40	81.8	24.2	—	
2. Body height (cm)	40	170.5	9.4	0.43	—
3. FAEO (sway area in cm^2)	40	1.4	1.4	0.05	0.28
4. FAEC (sway area in cm^2)	40	1.3	0.8	0.08	-0.03
5. FTEO (sway area in cm^2)	40	4.6	2.0	0.13	0.05
6. FTEC (sway area in cm^2)	40	7.6	4.8	0.28	0.12
